# Detection of Anaphylaxis Through the Cutaneous Barrier

**DOI:** 10.1007/s11882-025-01218-5

**Published:** 2025-09-29

**Authors:** Anjali Sundar, Marc S. McMorris, Charles F. Schuler

**Affiliations:** 1 Mary H. Weiser Food Allergy Center, Ann Arbor, MI USA; 2 Division of Allergy and Clinical Immunology, Department of Internal Medicine, Ann Arbor, MI USA; 3 Department of Pediatrics, Ann Arbor, MI USA; 4 Graduate Program in Immunology, University of Michigan, Ann Arbor, MI USA

**Keywords:** Anaphylaxis detection, Oral food challenge, Atopic dermatitis, Transepidermal water loss, Skin barrier, Epithelial barrier, Cutaneous barrier, Tight junctions, Food allergy

## Abstract

**Purpose of Review:**

The capacity to rapidly and objectively detect impending anaphylaxis is a crucial unmet need in food allergy, as clinical impression remains the only means of anaphylaxis diagnosis. Changes in the cutaneous barrier during an allergic reaction might offer an objective, rapid, and accessible anaphylaxis detection method.

**Recent Findings:**

Changes in cutaneous temperature and skin permeability might serve as markers of anaphylaxis. Existing methods around facial thermography, cutaneous blood flow measurements, electrical impedance spectroscopy, and transepidermal water loss (TEWL) offer varied data as possible food anaphylaxis biomarkers. Further data is needed to validate these and other methods as a means to non-invasively detect anaphylaxis.

**Summary:**

This review describes key advances in anaphylaxis detection through the cutaneous barrier, most notably around skin barrier function in the context of atopic dermatitis and food allergy.

## Introduction

Anaphylaxis is a life-threatening reaction to an allergic trigger. These triggers are broad, and include foods, insect venom, medications, heat, exercise, and in some cases, the cause is idiopathic. The presentation of anaphylaxis may be variable according to age, with children often showing more cutaneous symptoms and elderly often demonstrating more cardiovascular symptoms [1–3]. There are also several procedures conducted by allergists that pose a risk of anaphylaxis such as rush and ultra-rush venom and allergen immunotherapy protocols, oral food challenges (OFCs), and drug challenges [4]. Studies surrounding the detection of anaphylaxis through the skin barrier have focused on food-related anaphylaxis in the pediatric population, likely because of the more predictable reaction pattern in children with a known food trigger.

Food allergy is a highly prevalent disease, affecting over 10% of the US population [5]. Oral food challenges (OFCs) are the gold standard in food allergy diagnosis, but such challenges impose cost and time barriers alongside a risk of anaphylaxis [6]. Additionally, allergists experience barriers to performing OFCs, including training, experience, time, and effort [7]. The use of OFCs in clinical practices is not widespread, in part due to allergists’ concerns as well as reported safety concerns, parental anxiety, and aspects of identifying anaphylaxis in young children [8]. Adherence to guidelines for treating anaphylaxis varies during OFCs, further complicating OFC performance [9, 10]. Despite these challenges, OFCs conducted in controlled settings by allergists are generally safe, with the majority of reactive pediatric challenges developing only mild symptoms [9]. Indeed, OFCs carry major benefits for children, such as reduced costs and improved food-related quality of life [11, 12]. A means for food anaphylaxis detection, particularly in children, could greatly improve utilization and safety of the OFC. A validated anaphylaxis detection method may also be applicable to anaphylaxis as a result of other triggers. This review therefore describes major advances in anaphylaxis detection through the skin barrier.

### Current Strategies to Improve the Safety of Oral Food Challenges

There have been numerous efforts to make OFCs safer. One study employed a risk-stratified protocol based on milk avoidance, asthma, and milk-specific IgE levels to reduce the risk of severe reactions during initial milk OFCs [13]. Additionally, skin prick tests and serum IgE levels might help guide decision making when offering oral food challenges to patients. In one study of infants, peanut and sesame skin prick tests with a wheal of 8 mm or greater and egg skin prick tests with a wheal of 5 mm or greater had a 95% positive predictive value for challenge-proven food allergy [14]. Additionally, serum IgE of 34 kUa/L or greater for peanut and 1.7 kUa/L or greater for egg had a 95% positive predictive value for challenge-proven food allergy [14]. The authors concluded that using this data might help avoid conducting unnecessary and potentially dangerous oral food challenges [14]. New tests, including basophil activation testing and epitope analysis may provide more accurate diagnosis [15], but use of these methods remains unstandardized and require further development.

The ability to predict anaphylaxis would be of great value in standardizing and enhancing the safety of OFCs. The likelihood of anaphylaxis appears to increase with age, may be more common in adolescents, and is most likely in relation to food triggers [16]. Early recognition of the signs and symptoms of anaphylaxis in an infant is critical given this non-verbal age may be associated with more subtle signs of an allergic reaction [17]. A consensus to standardize food challenge procedures has been offered [18]; however, severity scores for food-induced anaphylaxis are not standardized, which may delay the administration of epinephrine [19]. Simplifying anaphylaxis decision-making for healthcare professionals using severity grading systems may address this issue [19].

### Anaphylaxis Monitoring as a Means to Mitigate Fear and Costs

Parental fear and anxiety around a potential reaction are major barriers to greater use of oral food challenges [10]. A successful food challenge decreases maternal anxiety surrounding allergic reactions, and trust in a child’s physician seems to promote maternal comfort in having a child participate in a food challenge [20]. One study showed that fear of the unknown was a common concern among mothers [21]. Other studies suggest that mothers reported increased anxiety on the day of a food challenge, but even positive food challenges are associated with improved food-related quality of life [22, 23]. Furthermore, children with food allergy may be at risk for posttraumatic stress, especially those with a history of anaphylaxis [24]. There are also economic considerations in treating allergic reactions. A means of detecting anaphylaxis may allow for earlier administration of epinephrine, potentially reducing the risk of severe or biphasic anaphylaxis and the need for emergency room visits [25, 26]. Access to epinephrine autoinjectors is also limited worldwide and these devices are expensive, placing a high burden on the treatment of systemic allergic reactions [27]. The use of a monitoring device might mitigate barriers around OFCs for patients and families, including cost.

### Atopic Dermatitis and Food Allergy

The link between atopic dermatitis and food allergy suggests there is value in studying the epithelial barrier in this context. There is increasing evidence to suggest that a disrupted skin barrier is key to the development of food allergy, suggesting a possibility of shared epithelial barrier dysfunction between atopic dermatitis and food allergy [28, 29]. Epicutaneous sensitization to food allergens occurs through epithelial barrier dysfunction, changes in the skin microbiome, and immune dysregulation [29]. Filaggrin loss-of-function mutations are associated with the development of food allergy in older children through eczema, as well as food allergy sensitization during early childhood [30, 31]. Palmar hyperlinearity in children is associated with filaggrin mutation and sensitization to egg [32]. In one study, children undergoing a double-blind, placebo-controlled food challenge with at least one loss-of-function variant of the filaggrin gene were 1.5 times more likely to be clinically food reactive [33]. In a prospective study focused on nonlesional skin, children with atopic dermatitis and food allergy had increased TEWL, low filaggrin breakdown products, changes in stratum corneum lamellar bilayer structure, and an altered fatty acid skin mix when compared to children with atopic dermatitis without food allergy [34]. These findings suggest that children with food allergy and atopic dermatitis have abnormalities in the stratum corneum which may make them more susceptible to the development of food allergy. Finally, one study showed that mutations in the SPINK5 skin barrier gene is associated with IgE-mediated food allergy in children [35]. These studies demonstrate that the epithelial barrier is intrinsically linked to food allergy.

### The Epithelial Barrier and Allergic Disease

**Fig. 1:**
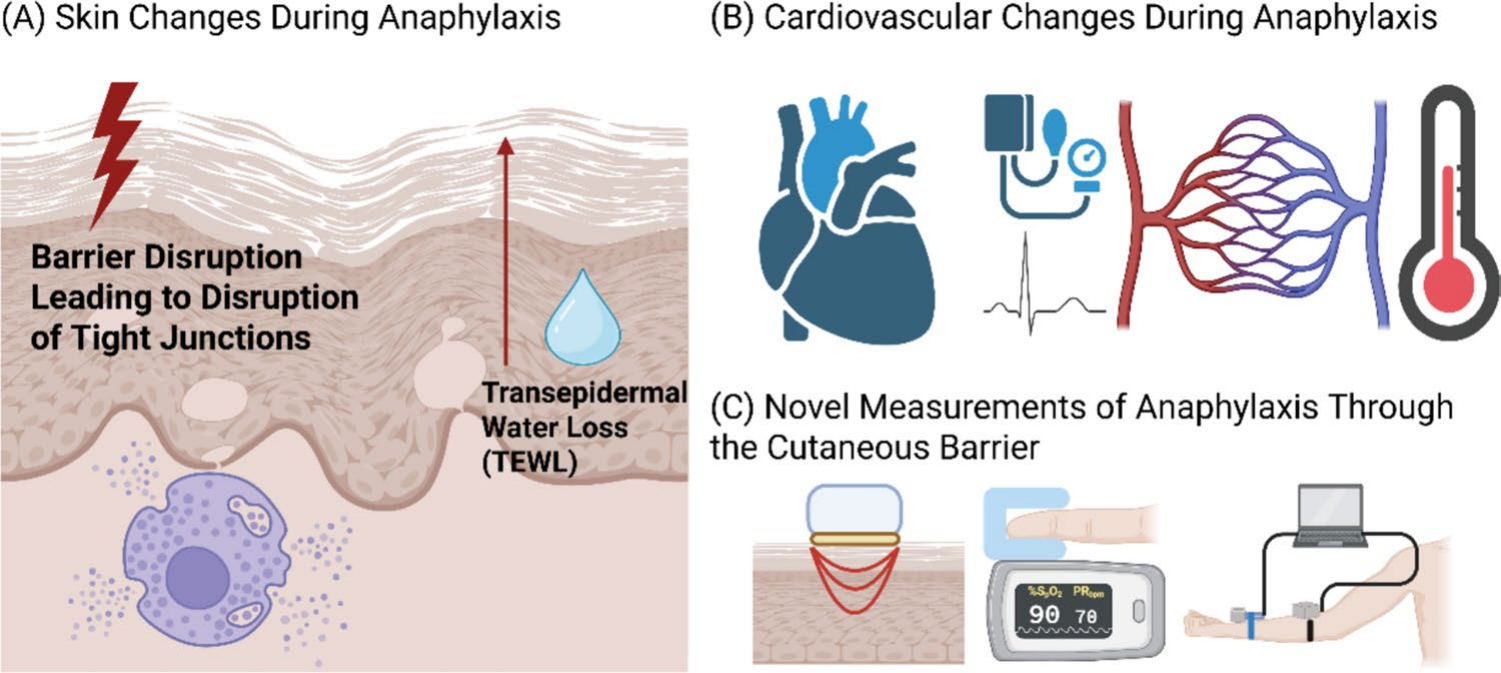
Summary of skin and cardiovascular changes during anaphylaxis and current methods to detect anaphylaxis through the cutaneous barrier. (**A**) Skin barrier disruption during anaphylaxis leading to disruption of tight junctions, increase in transepidermal water loss (TEWL), and mast cell degranulation; **B**) Cardiovascular changes during anaphylaxis including hypoxia, hypotension, tachycardia, changes in vascular permeability, and decreased core body temperature; **C**) Novel measurements of anaphylaxis through the cutaneous barrier including electrical impedance spectroscopy, cutaneous blood flow measurements, and TEWL measurements using open and closed-chamber devices. Created in BioRender. Sundar, A. (2025) https://BioRender.com/9g70xdf

On a broader level, the epithelial barrier has been linked to various allergic diseases, suggesting a potential target for the diagnosis and treatment of allergic diseases (Fig. 1a). The dysfunctional epithelial barrier allows allergens, irritants, and microbes to penetrate the skin and induce a type 2 immune response [36]. Increased airway permeability is a major component of chronic airway inflammation, as it predisposes individuals to be exposed to exogenous allergens, microbes, and pollutants [37]. One review of epithelial structural proteins highlighted that the keratin cytoskeleton and desmosomes are important to maintain the epithelial barrier, and dysfunction of these proteins can lead to several diseases [38]. As a result, epithelial barrier repair strategies including use of emollients, biologics, environmental control, and altering the microbiome are approaches to prevent or intervene on allergic disease [39].

Tight junction permeability on various levels has also been linked to the pathophysiology of many disease processes. Defects in tight junctions can have hereditary or secondary causes [40]. In one study, ovalbumin-sensitized rats had an increase in intestinal permeability due to changes in the tight junction proteins [41]. Junctional adhesion molecule (JAM-A), a cell surface protein, regulates cell migration, epithelial and paracellular permeability, as well as inflammation and proliferation in the intestines [42, 43]. One study showed altered intestinal epithelial integrity from reduced JAM-A expression as a result of mast cell degranulation and release of tryptase, which could provide some explanation for the mechanism of irritable bowel syndrome [44]. Another study showed that chymase released from mast cells has an effect on human small airway epithelial cells through regulation of the remodeling of the extracellular matrix that occurs in diseases such as asthma [45]. Mast cell proteases such as tryptase and chymase have been found to contribute to impaired bronchial epithelial responses during asthma exacerbations by decreasing the expression of epithelial barrier proteins [46]. Research on the roles of gap and tight junctions is needed to better understand endothelial permeability in anaphylaxis, in order to improve how we treat allergic reactions [47].

### Physiologic Changes During Anaphylaxis

A number of physiologic changes occur with an allergic reaction, including a change in vascular permeability, temperature, and respiratory function and several of these may be used as markers or predictors of impending anaphylaxis. Vital sign changes that occur with anaphylaxis include hypotension, tachycardia, and hypoxia [48]. From a cardiovascular hemodynamic perspective, venous return decreases, stroke volume decreases, and heart rate increases during food-induced allergic reactions [49] (Fig. 1b). Recent evidence has shown that diastolic blood pressure increases early in allergic reactions, which may serve as a predictor of oral food challenge outcomes [50]. A decrease in serum protein and human serum albumin levels has been noted during anaphylaxis and can be used as indirect measurements of extravasation in the human body and of anaphylaxis severity [51]. Histamine and platelet activating factor modulate vascular permeability by leading to the release of endothelial nitric oxide, which in turn leads to vasodilation and disruption of adherence junctions between endothelial cells [52]. Local allergic responses have been measured in mice using dye extravasation of the ear, as a marker of change in vascular permeability [53]. In vitro studies showed that overexpression of regulator of calcineurin 1 (Rcan1), which is a negative regulator of mast-cell degranulation, promotes barrier integrity [54]. It was found that endothelial Rcan1 is increased in response to anaphylaxis as a mechanism of strengthening the endothelium and mice who were Rcan-1 deficient had a more severe response to histamine [54]. The TNF-like weak inducer of apoptosis (TWEAK) and fibroblast growth factor-inducible molecule 14 (Fn14) have been studied as mediators of vascular permeability and anaphylaxis [55]. TWEAK and Fn14 were found to have increased expression in passive and active anaphylaxis [55]. Furthermore, administration of histamine and platelet activating factor was found to increase Fn14 expression in lung and endothelial cells [55]. The small GTPase RhoA and stimulation of the serine-threonine kinase ROCK mediates histamine-induced vascular leakage by disrupting the continuity of the endothelial barrier [56]. This suggests that inhibition of RhoA or ROCK may be targets for diseases that involve vascular leakage [56]. Several of the physiological changes that accompany anaphylaxis are associated with markers that can be measured as a means of detecting an impending allergic reaction.

Temperature has been studied in comparison to other physiologic changes during allergic reactions. A 1954 paper found that a fall in rectal temperature in mice is associated with anaphylactic shock [57]. In a study of peripheral tissue temperature monitoring, patients with anaphylaxis experienced a rapid increase in foot temperature that correlated with severity of reaction and predicted cardiovascular collapse [58]. A literature review focused on temperature as a component of perioperative anaphylaxis suggested that anesthesia induced body temperature changes are related to anaphylaxis [59]. When sweating was inhibited in subjects and TEWL was measured, an increase in TEWL was seen with an increase in skin temperature [60]. One study looked at a climate chamber in which ambient temperature and relative humidity were independently controlled, and found that there was a higher correlation between TEWL and ambient temperature, compared with relative humidity [61]. Skin temperature was also more closely related to ambient temperature, than relative humidity [61]. Also, skin surface hydration appeared to be affected by both ambient temperature and relative humidity [61]. In one study, local heating of the skin did not affect vasoconstriction in response to norepinephrine, independent of nitric oxide and differences in baseline skin blood flow, suggesting that changes in local temperature of the skin are not solely responsible for changes in cutaneous vasoconstriction [62]. Temperature changes during anaphylaxis can be reflected by skin property changes, further supporting the role of studying the cutaneous barrier as a means of detecting an allergic response.

### Current Methods of Detecting Anaphylaxis

Some current methods of detecting anaphylaxis include facial thermography, measurement of peripheral perfusion, and electrical impedance spectroscopy [63–65] (Fig. 1c). In one study, facial thermography was able to detect a rise in nasal temperature before objective symptoms arose in patients who had a positive food challenge [63]. A single-center observational study showed that cutaneous blood flow, measured with a laser doppler monitor, increased in patients who had symptoms during an oral food challenge [64]. In patients who had no reaction during oral food challenge, the cutaneous blood flow remained at baseline [64]. Electrical impedance, assessed with spectroscopy, has been found to be reduced in mice who had a damaged epidermal barrier, along with an increase in TEWL [65]. Studies have demonstrated that electrical impedance spectroscopy is able to discriminate between healthy skin and atopic dermatitis [66]. Electrical impedance spectroscopy has also been found to be less affected by daily routine activities such as coffee intake and intense exercise, compared to TEWL [67]. Electrical recordings of skin hydration are not measuring water directly, however, which may affect whether such measurements are more or less accurate than a given TEWL measurement [68].

### TEWL as a Marker of Skin Barrier Permeability

Monitoring TEWL as a marker of skin barrier permeability may serve as a non-invasive way to predict anaphylaxis [69] (Fig. 1c). In one study, skin tape strip samples from children with atopic dermatitis showed that there are certain proteins that are correlated with TEWL and allergic sensitization which can be non-invasively assessed [70]. Several studies on TEWL have focused on various compounds that may serve as modulators of TEWL by affecting the skin barrier and there is increasing evidence showing that TEWL rises with a disrupted skin barrier [71, 72]. Similarly, studies have shown that improved skin hydration has been correlated with reduced TEWL [73–75]. Another study evaluated measurements of stratum corneum hydration via capacitance, dermal water via tissue dielectric constant, and TEWL as different means to measure skin tissue water [76]. The study demonstrated that stratum corneum hydration is directly linked to dermal tissue water levels, but TEWL was not found to have this association at all sites of the body [76]. There have also been studies to compare TEWL among different skin tones, which have shown large variability, and, therefore, no definitive conclusions have been made [77–81].

The impact of environmental factors on skin properties influences our understanding of the epithelial barrier. A recent literature review of the impact of airborne pollution on atopic dermatitis found that airborne pollutants contribute to skin epidermal barrier dysfunction [82]. Studies have shown that TEWL can be increased in active and passive smokers, as well as those with exposure to particulate matter, airborne formaldehyde, and airborne volatile organic compounds [83–86].

A study on the various methods for measuring water content in the skin discusses that TEWL can be measured with open-chamber methods, semi-open methods, and closed-chamber methods [68]. A recent study showed that TEWL rise was found to occur 38 min earlier than clinically evident anaphylaxis during oral food challenges [87, 88]. A TEWL rise of 1 g/m^2^/h was found to be 100% sensitive for anaphylaxis [87]. TEWL did not rise in non-reactive oral food challenges with a high predictive specificity of 96%, indicating that TEWL can be used as a specific monitor to detect food related anaphylaxis [87]. Measurements can vary among anatomical sites and optimal measurements require controlled conditions with regards to environmental humidity, temperature, and airflow [69]. A wearable closed-chamber TEWL device has been developed for continuous monitoring of skin water vapor flux and has been designed to distinguish between insensible and sensible water loss [89]. The closed-chamber design would ideally reduce variability in measurements that may be problematic in open-chamber designs [89]. Initial studies have been performed using closed-chamber devices, but more research is needed to evaluate their efficacy in predicting anaphylaxis.

## Conclusions

In conclusion, the cutaneous barrier is linked to allergic disease and disruption of this barrier is a key component of the pathophysiology of anaphylaxis. Early detection of anaphylaxis through the cutaneous barrier may mitigate the risk of allergic reactions both in clinical and non-clinical settings, lending value to this research area. Thermography and electrical impedance spectroscopy have been studied previously, but have not garnered sufficient evidence as reliable means of anaphylaxis detection. One group is studying smartwatches and artificial intelligence for remote monitoring of anaphylaxis [90]. TEWL has thus far shown the most mature data in predicting food anaphylaxis in a clinical setting [88]. Future work is needed to validate a method of detecting anaphylaxis through the cutaneous barrier so this may eventually become standard of care.

## Key References

- Leung, D.Y.M., E. Berdyshev, and E. Goleva, *Cutaneous barrier dysfunction in allergic diseases.* J Allergy Clin Immunol, 2020. 145(6): p. 1485-1497.
  - This is a key mansucript that highlights the role of the cutaneous barrier in allergic diseases. The paper discusses how cutaneous barrier dysfunction plays a central role in the pathophysiology of, not only, atopic dermatitis, but also may be linked to asthma, food allergy, and allergic rhinitis.
- Ruiz-Garcia, M., et al., Cardiovascular changes during peanut-induced allergic reactions in human subjects. J Allergy Clin Immunol, 2021. 147(2): p. 633-642.
  - This manuscript demonstrates the cardiovascular changes that occur in humans during anaphylactic reactions to peanut. Changes include a decrease in venous return and stroke volume and an increase in heart rate. The study also concludes that an inability to compensate for these changes may be associated with more severe reactions.
- Schuler, C.F., et al., Transepidermal water loss rises before food anaphylaxis and predicts food challenge outcomes. J Clin Invest, 2023. 133(16).
  - This manuscript highlights the role of transepidermal water loss (TEWL) in detecting anaphylaxis during oral food challenges. This study demonstrated that TEWL rise occurred 38 minutes earlier than clinically evident anaphylaxis and TEWL did not rise in non-reactive oral food challenges. TEWL was found to be both highly specific and sensitive for anaphylaxis. This study offers promising data that TEWL can be used as a means to detect anaphylaxis non-invasively through the cutaneous barrier.
- Sivakumar, A.D., et al., WASP: Wearable Analytical Skin Probe for Dynamic Monitoring of Transepidermal Water Loss. ACS Sens, 2023. 8(11): p. 4407-4416.
  - This manuscript highlights the role of a closed-chamber device to address environmental fluctuations that may impact the reliability of TEWL readings using an open-chamber device.

## Data Availability

No datasets were generated or analysed during the current study.
